# Exceptionally Long Recurrence‐Free Survival in a Male Patient With Fibrolamellar Hepatocellular Carcinoma

**DOI:** 10.1155/crhe/7445319

**Published:** 2026-02-08

**Authors:** Elizabeth C. Townsend, Disha Sharma, Harish Gopalakrishna, Maria Mironova, David E. Kleiner, Theo Heller

**Affiliations:** ^1^ Medical Scientist Training Program, University of Wisconsin School of Medicine and Public Health, Madison, Wisconsin, USA, uwm.edu; ^2^ Department of Gastroenterology, University of Maryland Medical Center, Baltimore, Maryland, USA, umaryland.edu; ^3^ Digestive Diseases Branch, National Institute of Diabetes and Digestive and Kidney Diseases, National Institutes of Health, Bethesda, Maryland, USA, nih.gov; ^4^ Liver Diseases Branch, National Institute of Diabetes and Digestive and Kidney Diseases, National Institutes of Health, Bethesda, Maryland, USA, nih.gov; ^5^ Department of Pathology, National Cancer Institute, National Institutes of Health, Bethesda, Maryland, USA, nih.gov

## Abstract

Fibrolamellar hepatocellular carcinoma (FLHCC) accounts for less than 1% of all primary liver cancers. Due to an absence of known risk factors, most individuals are diagnosed at advanced stages of disease. Subsequently, these tumors carry a 30%–50% mortality rate and a 33%–100% recurrence rate in those treated with curative intent. Here, we describe an incidental discovery of an FLHCC tumor in a young male patient, who following surgical resection has remained recurrence free for over 11 years. This exceptionally long recurrence‐free survival highlights the advantage of early‐stage detection, the benefit of complete tumor resection, and further underscores the importance of regular postresection cancer surveillance.

## 1. Introduction

Fibrolamellar hepatocellular carcinoma (FLHCC) is a rare primary liver cancer which accounts for less than 1% of all primary liver cancers [1]. Compared to hepatocellular carcinoma, FLHCC tends to involve males and females equally, affects younger patients (10–35 years of age), and those without underlying liver disease [2, 3]. In the absence of any known risk factors, serum biomarkers, and typical “malignancy” symptoms, roughly 60%–70% of individuals with FLHCC are diagnosed at advanced stages of disease [2, 4, 5]. For those without multifocal metastases, extensive nodal spread, or major vessel involvement, surgical resection remains the only potentially curative treatment. However, after resection, recurrence rates remain as high, with 5‐year recurrence‐free survival of 20%–45% and 5‐year overall survival of roughly 50%–60% [2, 5–7].

Here, we present a rare presentation of FLHCC, an incidental discovery of an early‐stage tumor in a male patient, who following surgical resection remains recurrence‐free at 11 years. This unusually long recurrence‐free survival highlights the importance of complete tumor resection and regular postresection cancer surveillance.

## 2. Case Report

A 31‐year‐old male with a medical history of Graves’ disease presented to his primary care physician with nonspecific respiratory symptoms, including cough, productive sputum, myalgias, and early satiety. A computed tomography (CT) scan of the chest with upper cuts of the abdomen incidentally revealed a large liver mass. At presentation, the patient denied any abdominal complaints, change in bowel habits, weight loss, or family history of liver‐related cancers. Physical examination including vital signs was unremarkable besides a liver palpable below the right costal margin, with a span of 14 cm. His alanine transaminase was notably elevated to 55 U/L, and gamma‐glutamyl transferase was high at 120 U/L. The remainder of his liver function tests was normal, including a total bilirubin of 0.3. Labs were also notable for an elevated erythrocyte sedimentation rate of 30, with a normal alpha fetoprotein (AFP) of 1.1 ng/mL. Chronic liver disease evaluation including workup for all viral hepatitis, autoimmune, and hereditary conditions was negative.

A subsequent abdominal CT with contrast confirmed a heterogeneous 10‐cm mass encompassing the left lobe, with multiple central scars and calcifications, and no evidence of metastasis (Figure 1(a)). An abdominal magnetic resonance imaging (MRI) also displayed the large 9.5‐cm mass involving the left lobe of the liver extending medially and displacing the stomach to the left. On T2 images, the mass appeared hyperintense and hypervascular with an intact capsule (Figure 1(b)). Esophagogastroduodenoscopy and colonoscopy performed to evaluate primary malignancy were unremarkable. A positron emission tomography scan ruled out metastasis. Collectively, the atypical clinical presentation, absence of an AFP elevation, and imaging findings strongly suggested FLHCC.

Figure 1Imaging of the liver lesion. (a) Abdominal CT with contrast, late arterial phase, demonstrating a heterogeneous 10‐cm mass encompassing the left hepatic lobe, with multiple central scars and calcifications. (b) Abdominal T2‐weighted fat suppressed MRI, coronal section illustrating the heterogeneous mass with the hypointense central scar.

**Figure:**
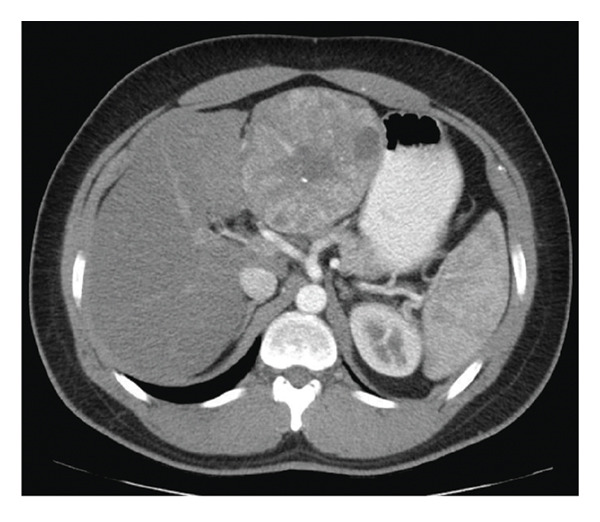
(a)

**Figure:**
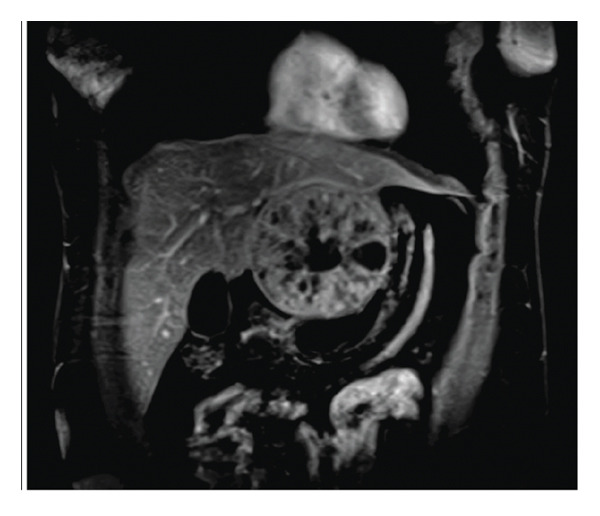
(b)

The patient underwent a successful complete resection of the tumor, without complication and without need for subsequent chemotherapy. Pathologic assessment of the lesion revealed large polygonal cells arranged in narrow trabeculae with a high nuclear‐to‐cytoplasmic ratio and nuclear atypia with prominent nucleoli (Figures 2(a) and 2(b)). Although there were no thick, fibrous lamellae, there was significant focal collagen deposition between cords of tumor cells. Immunostains for CD68 (KP‐1) and glypican 3 were negative. Overall, the findings on pathology most closely aligned with a well‐differentiated FLHCC. Regional lymph nodes were also evaluated, and all were negative for malignancy.

Figure 2Pathology of the liver lesion. (a) Low‐power hematoxylin and eosin (H&E) stain and (b) high‐power H&E stain showing large polygonal cells arranged in narrow trabeculae with significant collagen deposition between cords of tumor cells. Tumor cells display a high nuclear‐to‐cytoplasmic ratio and nuclear atypia with prominent nucleoli.

**Figure:**
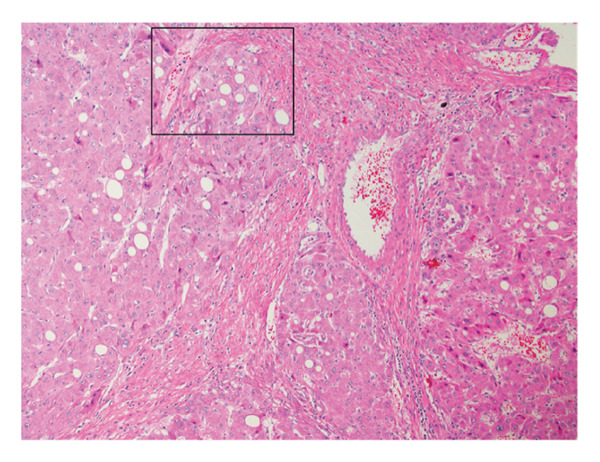
(a)

**Figure:**
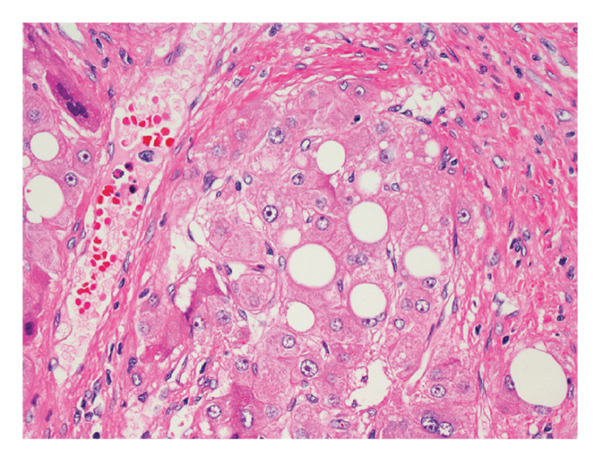
(b)

**Figure 3:**
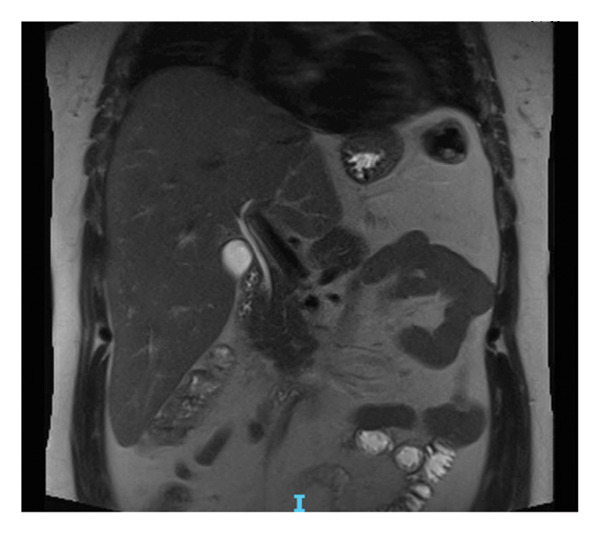
Imaging confirming the absence of recurrence 11 years following resection. Abdominal T2‐weighted MRI, coronal section.

He subsequently underwent regular annual surveillance with MRI and AFP. As of the writing of this report, he has remained free of recurrence at 11 years (Figure 3).

## 3. Discussion

Early identification of FLHCC is a challenge. Unlike classic hepatocellular carcinoma (HCC), which presents in older individuals, often 50–70 years of age, following decades of liver disease (e.g., metabolic liver disease and hepatitis B or hepatitis C infection), FLHCC generally presents in adolescents and young adults, 10–30 years of age, without any underlying liver condition [2]. Due to the rarity of the cancer, absence of biomarkers, absence of clear risk factors, and tendency for the cancer to occur in younger individuals, there are no recommendations to screen the general population for FLHCC. If not for undergoing a CT to evaluate his unrelated respiratory symptoms, it is likely his tumor would have continued to go undetected for quite some time. Most individuals with FLHCC initially present with nonspecific symptoms, including abdominal pain, distention, a palpable mass, weight loss, malaise, and/or symptoms related to metastasis [8]. At diagnosis, tumors are confined to the liver in roughly 30% of cases, have spread to regional lymph nodes in roughly 60%, and have metastasized to distant sites in 20%–30% of cases [4, 5, 8, 9]. The most common sites of metastasis include the lungs, peritoneum, gallbladder, and adrenal glands [8–10].

Diagnosis of FLHCC is generally based on imaging findings and clinical presentation. Although not diagnostic, negative AFP levels can support the diagnosis. On contrast CT, FLHCC tumors tend to be a large, well‐defined, lobulated mass with heterogeneous arterial phase enhancement [10]. As in this case, over 70% of FLHCC contain a central stellate scar with internal calcifications. Although not pathognomonic, a large (> 2 cm) calcified scar with radiating fibrotic bands is a useful diagnostic feature. On MRI, FLHCC is usually hypointense on T1‐weighted imaging and hyperintense on T2‐weighted imaging with a hypointense central fibrous scar on both T1‐ and T2‐weighted imaging [10]. Although the role of nuclear medicine in the diagnostic evaluation of FLHCC has not been fully defined, fludeoxyglucose‐18 positron emission tomography (FDG PET) can be useful to evaluate for extrahepatic metastasis and cancer staging once a liver lesion is detected [10].

FLHCC receives its name from its distinct histopathology, and its pathology remains the gold standard for confirming its diagnosis. On gross exam, FLHCC tumors are typically large, tan‐colored, well‐circumscribed, firm masses, and the central stellate scar can be macroscopically visible in as many as 75% of cases [10]. Microscopically, FLHCC classically contains thick, intratumoral lamellar collagen bands that separate chords of large polygonal cells [11]. These cells display abundant eosinophilic cytoplasm, large vesiculated nuclei, and large nucleoli. Roughly 90% of FLHCC cases are also positive for CD68 on immunostaining [12, 13]. This patient’s pathology was somewhat atypical in which the areas of dense collagen deposition were not arranged in thick lamellar bands and CD68 immunostaining was negative. Although these atypical features complicated the histologic diagnosis, the tumor’s appearance on hematoxylin and eosin stain was considered most consistent with FLHCC. Though we were unable to perform molecular characterization on this tumor, this patient’s initial diagnosis reports have found almost all FLHCC tumors to be characterized by the DNAJB1‐PRKACA oncogenic driver fusion gene [14, 15].

Surgical resection with negative margins remains the only potentially curative treatment for FLHCC [1, 8]. Liver transplant can also be considered for those with large or otherwise not surgically amenable to hepatectomy without nodal involvement [1, 2, 8, 16]. Across studies, patients treated with curative intent, including resection, transplant, or radiofrequency ablation, have a 5‐year overall survival of 50%–70% [2, 6, 16, 17]. Recurrence after resection is very common, ranging from 33% to 100% with median recurrence‐free survival rates of 20–48 months [6, 7]. For the 20%–25% of patients with unresectable disease either due to multifocal metastases, extensive nodal spread, and/or major vessel involvement, the 5‐year survival is < 5% with a median survival of < 12 months [4, 7, 8, 18].

Systemic therapy with chemotherapy (e.g., gemcitabine, cisplatin, 5‐fluorouracil, interferon, and oxaliplatin‐based regimens) or targeted agents (e.g., sorafenib, mTOR inhibitors, or antiestrogenic agents) can be an appropriate option for patients with unresectable tumors [1, 19, 20]. However, currently there is no consistently effective systemic agent, and there are unfortunately no trials supporting the benefit of one regimen over another [19–22]. There has been increasing research into targeting oncogenic pathways downstream of DNAJB1‐PRKACA [23–25]. Neratinib, a pan‐HER signaling inhibitor targeting the HER2 signaling pathway, which is upregulated in DNAJB1‐PRKACA–positive FLHCC tumors, combined with immunotherapy, has shown promise in a Phase II trial [23].

The unusually long recurrence‐free survival of this case highlights both the advantage of early‐stage detection and the benefit of complete tumor resection with clear margins. These features remain the best prognostic indicators for patient outcomes. Other features associated with better prognosis include fewer tumors at diagnosis, normal liver enzymes, normal AFP, no nodal involvement, absence of large vessel involvement, or metastasis [5, 7, 8, 18, 26, 27]. The influence of demographic features, such as gender, age, and race/ethnicity, on overall prognosis remains unclear [4, 8, 21].

Although classic HCC and FLHCC carry distinct risk profiles, the National Comprehensive Cancer Network (NCCN) does not discriminate between the two in its recommendations [28]. Their guidelines encourage imaging every 3–6 months for 2 years, followed by every 6–12 months following resection for HCC. Since those with FLHCC are at high risk for recurrence, it may be beneficial to survey these patients closer to every 3 months for 2 years and then every 6 months for another 3‐4 years, followed by every year. Although the length for ongoing surveillance for FLHCC is also not clear, continuing surveillance likely carries significant benefit. The vast majority of first recurrences occur within 4‐5 years after surgical resection [6, 7], yet there are rare reports of delayed recurrences occurring 6 or more years after resection [7]. Once detected, surgical resection of recurrent disease has been shown to confer significant survival benefit [6].

## Funding

No funding was received for this manuscript.

## Consent

Informed consent was obtained from the individual participant included in this study, and with their consent, they allowed the inclusion, processing, and presentation of their personal data.

## Conflicts of Interest

The authors declare no conflicts of interest.

## Data Availability

The data that support the findings of this study are available upon request from the corresponding author. The data are not publicly available due to privacy or ethical restrictions.

## References

[1] Glavas D. , Bao Q. R. , Scarpa M. et al., Treatment and Prognosis of Fibrolamellar Hepatocellular Carcinoma: A Systematic Review of the Recent Literature and Meta-Analysis, Journal of Gastrointestinal Surgery. (2023) 27, no. 4, 705–715, 10.1007/s11605-023-05621-z. 36797535 PMC10073062

[2] Eggert T. , McGlynn K. A. , Duffy A. , Manns M. P. , Greten T. F. , and Altekruse S. F. , Fibrolamellar Hepatocellular Carcinoma in the USA, 2000-2010: A Detailed Report on Frequency, Treatment and Outcome Based on the Surveillance, Epidemiology, and End Results Database, United European Gastroenterology Journal. (2013) 1, no. 5, 351–357, 10.1177/2050640613501507, 2-s2.0-85006217670. 24917983 PMC4040774

[3] El-Serag H. B. and Davila J. A. , Is Fibrolamellar Carcinoma Different From Hepatocellular Carcinoma? A US Population-Based Study, Hepatology. (2004) 39, no. 3, 798–803, 10.1002/hep.20096, 2-s2.0-1542724808. 14999699

[4] Aloysius M. M. , Iskander P. , Ahmed K. et al., Fibrolamellar Hepatocellular Carcinoma: An Epidemiologic and 5-Year Cancer Survival Assessment Based off SEER Data, Clinics and Research in Hepatology and Gastroenterology. (2023) 47, no. 7, 10.1016/j.clinre.2023.102162. 37307948

[5] Yamashita S. , Vauthey J. N. , Kaseb A. O. et al., Prognosis of Fibrolamellar Carcinoma Compared to Non-cirrhotic Conventional Hepatocellular Carcinoma, Journal of Gastrointestinal Surgery. (2016) 20, no. 10, 1725–1731, 10.1007/s11605-016-3216-x, 2-s2.0-84979547140. 27456016

[6] Groeschl R. T. , Miura J. T. , Wong R. K. et al., Multi-Institutional Analysis of Recurrence and Survival After Hepatectomy for Fibrolamellar Carcinoma: Hepatectomy for Fibrolamellar Carcinoma, Journal of Surgical Oncology. (2014) 110, no. 4, 412–415, 10.1002/jso.23658, 2-s2.0-84905708950. 24844420

[7] Stipa F. , Yoon S. S. , Liau K. H. et al., Outcome of Patients with Fibrolamellar Hepatocellular Carcinoma, Cancer. (2006) 106, no. 6, 1331–1338, 10.1002/cncr.21703, 2-s2.0-33644840201. 16475212

[8] Ang C. S. , Kelley R. K. , Choti M. A. et al., Clinicopathologic Characteristics and Survival Outcomes of Patients With Fibrolamellar Carcinoma: Data From the Fibrolamellar Carcinoma Consortium, Journal of Gastrointestinal Cancer. (2013) 6, no. 1, 3–9. PMC3597938 23505572

[9] Berkovitz A. , Migler R. D. , Qureshi A. et al., Clinical and Demographic Predictors of Survival for Fibrolamellar Carcinoma patients—A Patient Community, Registry-Based Study, Hepatology Communications. (2022) 6, no. 12, 3539–3549, 10.1002/hep4.2105. 36245434 PMC9701473

[10] Ganeshan D. , Szklaruk J. , Kundra V. , Kaseb A. , Rashid A. , and Elsayes K. M. , Imaging Features of Fibrolamellar Hepatocellular Carcinoma, American Journal of Roentgenology. (2014) 202, no. 3, 544–552, 10.2214/AJR.13.11117, 2-s2.0-84896735907. 24555590

[11] Kanel G. C. , Neoplasms and Related Lesions, Atlas of Liver Pathology, 2023, 4th edition, Elsevier, Atlas of Surgical Pathology Series.

[12] Graham R. P. , Yeh M. M. , Lam-Himlin D. et al., Molecular Testing for the Clinical Diagnosis of Fibrolamellar Carcinoma, Modern Pathology. (2018) 31, no. 1, 141–149, 10.1038/modpathol.2017.103, 2-s2.0-85040174730. 28862261 PMC5758901

[13] Ross H. M. , Daniel H. D. J. , Vivekanandan P. et al., Fibrolamellar Carcinomas are Positive for CD68, Modern Pathology. (2011) 24, no. 3, 390–395, 10.1038/modpathol.2010.207, 2-s2.0-79952195361. 21113139 PMC3292186

[14] Honeyman J. N. , Simon E. P. , Robine N. et al., Detection of a Recurrent *DNAJB1-PRKACA* Chimeric Transcript in Fibrolamellar Hepatocellular Carcinoma, Science. (2014) 343, no. 6174, 1010–1014, 10.1126/science.1249484, 2-s2.0-84896732099. 24578576 PMC4286414

[15] Graham R. P. , Jin L. , Knutson D. L. et al., DNAJB1-PRKACA is Specific for Fibrolamellar Carcinoma, Modern Pathology. (2015) 28, no. 6, 822–829, 10.1038/modpathol.2015.4, 2-s2.0-84930178233. 25698061

[16] El-Gazzaz G. , Wong W. , El-Hadary M. K. et al., Outcome of Liver Resection and Transplantation for Fibrolamellar Hepatocellular Carcinoma, Transplant International. (2000) 13, no. Suppl 1, S406–S409, 10.1007/s001470050372. 11112043

[17] Mavros M. N. , Mayo S. C. , Hyder O. , and Pawlik T. M. , A Systematic Review: Treatment and Prognosis of Patients With Fibrolamellar Hepatocellular Carcinoma, Journal of the American College of Surgeons. (2012) 215, no. 6, 820–830, 10.1016/j.jamcollsurg.2012.08.001, 2-s2.0-84869505053. 22981432

[18] Moreno-Luna L. E. , Arrieta O. , García-Leiva J. et al., Clinical and Pathologic Factors Associated With Survival in Young Adult Patients With Fibrolamellar Hepatocarcinoma, BMC Cancer. (2005) 5, no. 1, 10.1186/1471-2407-5-142, 2-s2.0-27744434462. PMC1310628 16259635

[19] Gras P. , Truant S. , Boige V. et al., Prolonged Complete Response After GEMOX Chemotherapy in a Patient with Advanced Fibrolamellar Hepatocellular Carcinoma, Case Reports in Oncology. (2012) 5, no. 1, 169–172, 10.1159/000338242, 2-s2.0-84860779811. 22666208 PMC3364045

[20] Fakih M. , A Case of Fibrolamellar Cancer With a Palliative Response and Minor Radiographic Regression With Erlotinib and Bevacizumab Combination Therapy, American Journal of Therapeutics. (2014) 21, no. 6, e207–e210, 10.1097/MJT.0b013e3182840fa6, 2-s2.0-84914110832. 23676344

[21] Kaseb A. O. , Shama M. , Sahin I. H. et al., Prognostic Indicators and Treatment Outcome in 94 Cases of Fibrolamellar Hepatocellular Carcinoma, Oncology. (2013) 85, no. 4, 197–203, 10.1159/000354698, 2-s2.0-84888234954. 24051705 PMC3913463

[22] Maniaci V. , Davidson B. R. , Rolles K. et al., Fibrolamellar Hepatocellular Carcinoma: Prolonged Survival With Multimodality Therapy, ournal of the European Society of Surgical Oncology. (2009) 35, no. 6, 617–621, 10.1016/j.ejso.2008.12.009, 2-s2.0-67349170783. 19144491

[23] Abou-Alfa G. K. , Mayer R. , Venook A. P. et al., Phase II Multicenter, Open-Label Study of Oral ENMD-2076 for the Treatment of Patients with Advanced Fibrolamellar Carcinoma, The Oncologist. (2020) 25, no. 12, e1837–e1845, 10.1634/theoncologist.2020-0093. 32154962 PMC8186410

[24] Abou-Alfa G. K. , Meyer T. , Do R. K. G. et al., Neratinib Alone or in Combination With Immune Checkpoint Inhibitors With or Without Mammalian Target of Rapamycin Inhibitors in Patients With Fibrolamellar Carcinoma, Liver Cancer. (2025) 14, no. 1, 58–67, 10.1159/000540290. 40144471 PMC11936434

[25] Gulati R. , Johnston M. , Rivas M. et al., β-Catenin Cancer-Enhancing Genomic Regions Axis is Involved in the Development of Fibrolamellar Hepatocellular Carcinoma, Hepatology Communications. (2022) 6, no. 10, 2950–2963, 10.1002/hep4.2055. 36000549 PMC9512470

[26] Lafaro K. J. , Eng O. S. , Raoof M. et al., A Prognostic Nomogram for Patients with Resected Fibrolamellar Hepatocellular Carcinoma, Hepatobiliary Surgery and Nutrition. (2019) 8, no. 4, 338–344, 10.21037/hbsn.2019.05.03. 31489303 PMC6700009

[27] McDonald J. D. , Gupta S. , Shindorf M. L. et al., Elevated Serum α-Fetoprotein is Associated With Abbreviated Survival for Patients With Fibrolamellar Hepatocellular Carcinoma Who Undergo a Curative Resection, Annals of Surgical Oncology. (2020) 27, no. 6, 1900–1905, 10.1245/s10434-019-08178-x. 31925595 PMC8456707

[28] Benson A. and D’Angelica M. , Hepatocellular Carcinoma, National Comprehensive Cancer Network Guidelines. (2024) 3.

